# Clinical and Therapeutic Characteristics of Hospitalized Patients with Advanced Lung Cancer in Najran, Saudi Arabia: A Retrospective Study

**DOI:** 10.7759/cureus.58602

**Published:** 2024-04-19

**Authors:** Ahmed M Badheeb, Hamoud Y Obied, Mohammed Al Suleiman, Mohammed A Qurayshah, Mohammad A Awad, Abdullah Abu Bakar, Batool Alwadai, Abdullah M Nasher, Islam A Seada, Nasher H Alyami, Abdelaziz A Aman, Faisal Ahmed, Abdulrahman Al Qasim, Mohamed Badheeb

**Affiliations:** 1 Oncology, King Khalid Hospital, Najran, SAU; 2 Cardiac Surgery, King Khalid Hospital, Najran, SAU; 3 Internal Medicine, King Khalid Hospital, Najran, SAU; 4 Ophthalmology, King Khalid Hospital, Najran, SAU; 5 Cardiothoracic Surgery, King Khalid Hospital, Najran, SAU; 6 Pathology and Hematology, Ministry of Health Holdings, Najran, SAU; 7 Urology, Ibb University, Ibb, YEM; 8 Internal Medicine, Yale New Haven Health, Bridgeport Hospital, Bridgeport, USA

**Keywords:** survival rate, pembrolizumab, chemoimmunotherapy, chemotherapy, advanced lung cancer, najran region, saudi arabia

## Abstract

Background: Lung cancer is one of the top causes of cancer deaths globally, including in Saudi Arabia. Although several prognostic markers have been established, the clinical features and outcomes of lung cancer in Saudi Arabia are not well understood. This study aimed to describe the clinical and therapeutic characteristics of advanced lung cancer in Najran, Saudi Arabia.

Method: A retrospective chart review of 44 patients diagnosed with advanced lung cancer between June 2018 and September 2021 and treated at the Oncology Center of King Khalid Hospital in Najran City, Saudi Arabia. The clinicopathological features, treatment used, response, and survival outcomes were collected and analyzed.

Result: The mean age was 69.3 ± 10.7 years, most of them (n = 35, 79.5%) were male and older than 70 years (n = 24, 54.5%). Adenocarcinoma was the most observed cancer (n = 35, 79.5%), followed by squamous cell carcinoma in six (13.6%). Most cases (n = 42, 95.5%) were in stage IV. Epidermal growth factor receptor (EGFR) mutations were positive in two (4.5%) cases and ALK mutation was positive in two (4.5%) cases. Metastasis to pleura with pleural effusion was the common presentation (n = 41, 93%). Chemotherapy was administered as the first line in 19 cases (43.2%) while 25 cases (56.8%) received chemoimmunotherapy. The commonest chemoimmunotherapy regimen used was carboplatin-pemetrexed-pembrolizumab in 16 (36.4%), followed by carboplatin-paclitaxel-pembrolizumab in 9 (20.5%) cases. The response to initial systemic therapy was as follows disease progression, stable disease, and complete remission in 10 (22.7%), 33 (75.0%), and 1 (2.3%), respectively. Median progression-free survival was 8.7 months (interquartile range (IQR): 5.7-11.4), and the median overall survival was 12.3 months (IQR: 11.1-13.4). Among the total documented 36 (81.8%) dead cases, disease progression was the main cause of death in 25 cases (56.8%). Using chemoimmunotherapy as the first-line therapy was associated with numerical survival improvement compared to using chemotherapy alone (HR: 0.75; 95% CI: 0.39-1.46) however, it was not statistically significant (p = 0.397).

Conclusion: In this study, the majority of lung cancer patients were male and over 70 years old. Adenocarcinoma was the most common histological type. Metastasis to pleura with pleural effusion was the common presentation. The most common treatment used was chemoimmunotherapy with a regimen of carboplatin-pemetrexed-pembrolizumab. Addressing the possible causes of delayed diagnosis of lung cancer is crucial for improved survival outcomes.

## Introduction

Lung cancer is one of the major global causes leading to death, with 2.2 million new cases diagnosed and 1.8 million deaths in 2020 [1]. The burden varies by country due to factors like smoking prevalence, environmental pollution, and dietary choices [2]. The most prevalent type of lung cancer is non-small-cell lung carcinoma (NSCLC), which accounts for 85% of cases [3]. Systemic therapy is recommended for disseminated disease. The type of systemic therapy depends on the histologic type, whether somatic genomic alterations are present that can be treated with targeted therapy, and performance status (PS) [4]. Various strategies are being explored to enhance survival in stage IV NSCLC patients, but comparing results across trials is challenging due to varying delivery times for the immune checkpoint inhibitors (ICIs) and eligibility criteria [5].

Lung cancer prevalence in Saudi Arabia has significantly increased in recent years, primarily due to increased cigarette smoking among both men and women [6]. In 2018, lung cancer was the fifth most common cancer-causing death among men and 12th among women in Saudi Arabia [7]. Furthermore, in 2018, 504 patients with lung cancer were recorded in Saudi Arabia, accounting for 3.2% of all newly diagnosed cases [8]. The incidence rate is expected to rise due to population expansion, including a sevenfold increase in older adults due to tobacco smoking [8]. Lung cancer incidence in Saudi Arabia is anticipated to nearly double, from 7.49% in 2020 to 13.87% in 2039 [9]. Even though the prevalence of lung cancer is on the rise, few previous studies have been conducted to analyze the pattern of lung cancer in our patients. Therefore, this study aimed to describe the clinical and therapeutic characteristics of advanced lung cancer in Najran, Saudia Arabia.

## Materials and methods

Study design, inclusion criteria, and exclusion criteria

A retrospective chart review of 44 patients diagnosed with advanced lung cancer between June 2018 and September 2021 and treated at the Oncology Center of King Khalid Hospital in Najran, Saudi Arabia. Adult patients aged 18 and above with advanced inoperable lung cancer were included. Pediatric patients and those with incomplete records were excluded.

Observation indicators and evaluation criteria

The initial lung cancer diagnosis used flexible fiberoptic bronchoscopy (FFB) or computed tomography (CT)-guided biopsy, with FFB used for core lung lesions and CT for peripheral ones. Mediastinoscopy and/or pleural biopsy were used when needed. Metastases were determined using body bone and CT scans. The pathological diagnosis of primary lung cancer was based on the new World Health Organization (WHO) categories of lung tumors [10]. Patients were categorized into four types: squamous cell carcinoma, adenocarcinoma, large cell carcinoma, and small cell carcinoma. Regular blood tests assessed liver and kidney function and were checked every three weeks. While CT scan and carcinoembryonic antigen (CEA), were evaluated every nine weeks. Therapeutic effectiveness was evaluated using the RECIST 1.1 criteria for solid tumors: A complete response (CR) is the disappearance of all target lesions; progressive disease (PD): an increase in the total diameters of target lesions by at least 20%, compared to the smallest cumulative measurement obtained throughout the research, or the formation of new lesions; stable disease (SD): insufficient shrinking to qualify for PR or sufficient rise to qualify for PD; progression-free survival (PFS): PFS is defined as the time beginning with the commencement of therapeutic intervention and extending until either the observed progression of the tumor or death due to any etiology. OS is defined as the period between the commencement of therapy and death from any cause [11]. Tissue-based next-generation sequencing (NGS) looking for 52 mutations was performed whenever adequate tumor tissue was available [12].

Data collection

Variables detected in this study include age, gender, clinical presentation, radiographic findings, associated symptoms, histopathological evaluation, tumor mutations using NGS testing, metastatic sites, management approach (chemotherapy, targeted therapy, first line, second line, salvage chemotherapy administration, and immunotherapy), response to treatment, current status (survive or died), and OS.

Statistical analysis

Quantitative data was reported using mean and standard deviation, while qualitative variables were reported using frequencies and percentages. Quantitative variables were tested using independent samples T-test or Mann-Whitney U, while qualitative variables were tested using chi-square or Fisher's exact, and survival probabilities were evaluated using Kaplan-Meier curves. Statistical significance was set at p < 0.05. Statistical analysis was done using IBM SPSS Statistics for Windows, Version 22 (Released 2013; IBM Corp., Armonk, New York, United States).

Ethical approval

The study was approved by the Ethics Research Committees of King Khalid Hospital (Code: KACST, KSA: H-I1-N-085), in compliance with the ethical standards outlined in the Declaration of Helsinki. Due to the anonymous retrospective nature of the study, written informed consent from the included patients was not required.

## Results

A total of 44 cases of advanced lung cancer were identified. The mean age was 69.3 ± 10.7 (range 45- 88 years), and most of them (n = 24, 79.5%) were male and older than 70 years (n = 24, 54.5%). Most cases were ECOG PS grades three in 28 (63.6%). Adenocarcinoma was observed in 35 (79.5%), followed by squamous cell carcinoma in six (13.6%) and two (4.5%) of cases were small cell carcinoma. Most cases (n = 42, 95.5%) were in stage IV. Metastasis to pleura with pleural effusion was the common presentation (n = 41, 93%). Comorbidities include hypertension, coronary artery disease, diabetes mellitus, and chronic obstructive pulmonary disease (COPD) in 25 (56.8%), 20 (45.5%), 19 (43.2%), and 14 (31.8%), respectively. NGS testing was conducted in 31 (70%.5) of cases. Epidermal growth factor receptor (EGFR) mutation was positive in two (4.5%) cases and KRAS mutation was positive in two (4.5%) cases. Metastasis to pleura with pleural effusion was seen in 41 (93%) cases. Additionally, lymph node metastasis (n = 27, 61.4%), and bone metastasis (n = 19, 43.2%) were the other common sites of metastasis. The incidence of metastasis to the opposite lung was seen in eight (18.2%) cases. The demographic and clinical characteristics of the 44 lung cancer patients are presented in Table 1.

**Table 1:** Pre-treatment characteristics of lung cancer patients *Some cases had multiple metastases. COPD: Chronic obstructive pulmonary disease; ECOG: Eastern Cooperative Oncology Group; EGFR: Epidermal growth factor receptor; ALK: Anaplastic lymphoma kinase gene rearrangements

Variables	N (%)
Age (year), mean ± SD	69.3 ± 10.7 (range 45-88)
Age more than 70 years	24 (54.5%)
Gender	
Male	35 (79.5%)
Female	9 (20.5%)
ECOG performance status	
1	3 (6.8%)
2	28 (63.6%)
3	9 (20.5%)
4	4 (9.1%)
Comorbidities	
Diabetes mellitus	19 (43.2%)
Hypertension	25 (56.8%)
COPD	14 (31.8%)
Coronary artery disease	20 (45.5%)
Pathology	
Adenocarcinoma	35 (79.5%)
Squamous cell carcinoma	6 (13.6%)
Small cell carcinoma	2 (4.5%)
Leiomyosarcoma	1 (2.3%)
Pathologic stage	
Stage III	2 (4.5%)
Stage IV	42 (95.5%)
EGFR mutation	
Negative	42 (95.5%)
Positive	2 (4.5%)
ALK mutation	
Negative	42 (95.5%)
Positive	2 (4.5%)
Metastasis location*	
Pleura with positive effusion	41 (93%)
Contralateral lung	8 (18.2%)
Bone	19 (43.2%)
Brain	5 (11.4%)
Liver	8 (18.2%)
Lymph node	27 (61.4%)
Other	14 (31.8%)

Treatment protocols and follow-up

Details of the treatment protocols the chemotherapy cycles and immunotherapy are given in Table 2. Chemotherapy was administered as the first line in 19 cases (43.2) while in 25 (56.8) cases received chemoimmunotherapy. The commonest chemoimmunotherapy regimen used was carboplatin-pemetrexed-pembrolizumab in 16 (36.4%), followed by carboplatin-paclitaxel-pembrolizumab in nine (20.5%) cases. Treatment was continued until intolerable toxicity, disease progression, physician decision, or patient withdrawal of consent, whichever occurred first. The response to initial therapy was as follows disease progression, stable disease, and complete remission in 10 (22.7%), 33 (75.0%), and 1 (2.3%), respectively. Second-line therapy was utilized in 28 (63.6%) cases. The main regimen used was Docetaxel in 10 (22.7%), followed by Gemcitabine in 9 (20.5%), and immunotherapy in 9 (20.5%) cases. The response to second systemic therapy was as follows disease progression, stable disease, and complete remission in 16 (36.4%), 27 (61.4%), and 1 (2.3%), respectively. Third-line chemotherapy was utilized in 13 (29.5%) cases. The main regimen used was Nivolumab in 10 (22.7%), followed by Vinorelbine in 3 (6.8%). The total mortality occurred in 36 cases (81.8%) and was described as disease progression in 25 cases (56.8%), and eight cases (18.2%) died due to other medical causes (Table 2).

**Table 2:** Treatment characteristics of advanced lung cancer patients

Variables	N (%)
First-line chemotherapy	
Carboplatin-pemetrexed-pembrolizumab	16 (36.4%)
Carboplatin-paclitaxel-pembrolizumab	9 (20.5%)
Carboplatin-pemetrexed	5 (11.4%)
Gemcitabine alone	3 (6.8%)
Carboplatin-gemcitabine	4 (9.1%)
Cisplatin-gemcitabine	3 (6.8%)
Osimertinib	2 (4.5%)
Alectinib	2 (4.5%)
Clinical outcomes	
Disease progression	10 (22.7%)
Stable disease	33 (75.0%)
Complete remission	1 (2.3%)
Second-line chemotherapy	
None (not fit)	16 (36.4%)
Docetaxel	10 (22.7%)
Gemcitabine	9 (20.5%)
Immunotherapy	9 (20.5%)
Clinical outcomes	
Disease progression	16 (36.4%)
Stable disease	27 (61.4%)
Complete remission	1 (2.3%)
Third-line chemotherapy	
None (not fit)	31 (70.5%)
Vinorelbine	3 (6.8%)
Immunotherapy	10 (22.7%)
Follow-up time (months), mean ± SD	11.9 ± 3.0 (range 2-21)
Outcome	
Live	8 (18.2%)
Death	36 (81.8%)
Cause of death	
Disease progression	25 (56.8%)
Other medical causes	8 (18.2%)
Unknown	3 (6.8%)

Survival analysis

**Figure 1:**
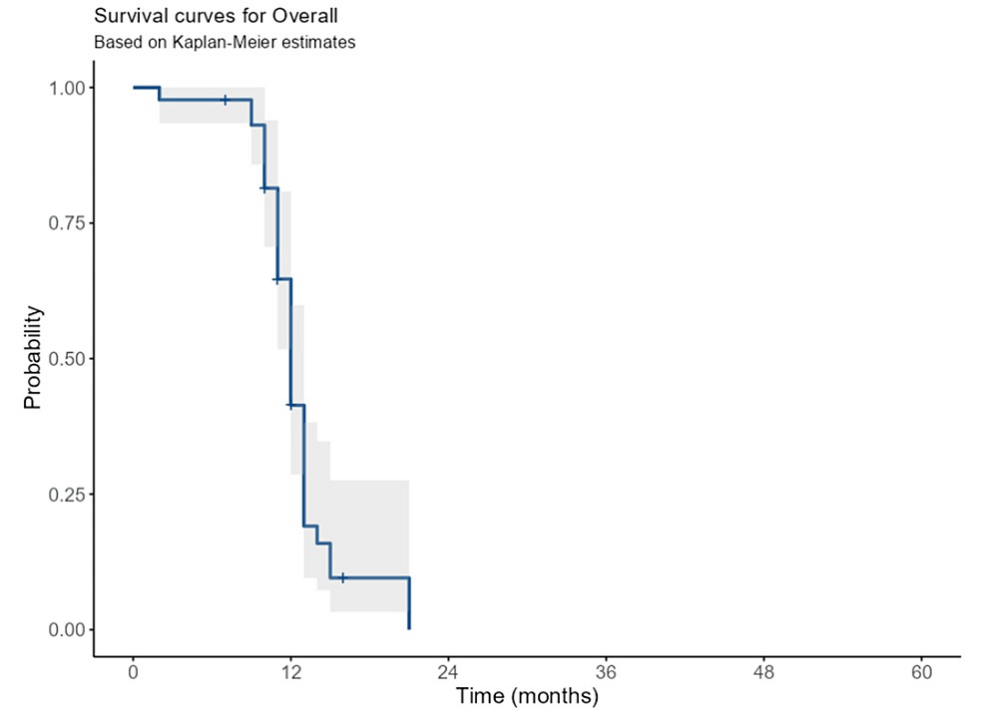
The overall survival of advanced lung cancer patients

Median progression-free survival was 8.7 months (interquartile range (IQR): 5.7-11.4), and the median OS was 12.3 months (IQR: 11.1-13.4) (Figure 1).

In patients receiving first-line treatment, the tumor objective response rate (ORR) was 43% (95% CI: 28%-65%), and disease progression was 30% (95% CI: 12%-77%). In patients receiving second-line treatment, the tumor ORR was 38.7% (95% CI: 23.3% -64), and disease progression was 42.2% (95% CI: 23.4%-76%). In Cox regression analysis, the risk of death for other adenocarcinoma cancer types was 1.60 (95% CI: 0.71-3.59) times compared to the adenocarcinoma type. However, it was not statistically significant (p = 0.254). The survival was improved for patients who received chemoimmunotherapy as the first-line therapy compared to patients who received chemotherapy alone (HR: 0.75; 95% CI: 0.39-1.46). However, it was not statistically significant (p = 0.397) (Table 3).

**Table 3:** Factors associated with survival in advanced lung cancer patients COPD: Chronic obstructive pulmonary disease; ECOG‐PS: Eastern Cooperative Oncology Group Performance Status; HR: Hazard ratio; EGFR: Epidermal growth factor receptor; ALK: Anaplastic lymphoma kinase gene rearrangements

Variables	Subgroup	N (%)	HR (95% CI)	p-value
Gender	Male	35 (79.5)	Reference group	0.997
	Female	9 (20.5)	1.00 (0.41-2.45)	
ECOG PS	1-2	32 (72.7)	Reference group	0.194
	3-4	12 (27.3)	0.61 (0.29-1.29)	
Pathology	Other pathology	9 (20.5)	Reference group	0.210
	Adenocarcinoma	35 (79.5)	0.60 (0.27-1.34)	
Age (year)	Mean ± SD	69.3 ± 10.7	1.00 (0.97-1.04)	0.798
Diabetes mellitus	No	25 (56.8)	Reference group	0.380
	Yes	19 (43.2)	1.36 (0.68-2.72)	
Hypertension	No	25 (56.8)	Reference group	0.388
	Yes	19 (43.2)	0.74 (0.37-1.47)	
COPD	No	30 (68.2)	Reference group	0.149
	Yes	14 (31.8)	0.58 (0.27-1.22)	
Coronary artery disease	No	24 (54.5)	Reference group	0.471
	Yes	20 (45.5)	0.78 (0.39-1.54)	
EGFR/ALK status	Negative	40 (90.9)	Reference group	0.454
	Positive	4 (9.1)	1.50 (0.52-4.30)	
First line therapy	Chemotherapy	19 (43.2)	Reference group	0.397
	Chemoimmunotherapy	25 (56.8)	0.75 (0.39-1.46)	
Second line therapy	Chemotherapy	35 (79.5)	Reference group	0.878
	Chemoimmunotherapy	9 (20.5)	1.07 (0.46-2.46)	

## Discussion

In this study, we describe the clinical and therapeutic characteristics of advanced lung cancer in Najran, Saudia Arabia. The main findings were that most patients were male and over 70 years old. Adenocarcinoma was the most common cell type. Metastasis to pleura with pleural effusion was the common presentation. Most cases received chemo-immuno-therapy and the commonest regimen was carboplatin-pemetrexed-pembrolizumab. The median progression-free survival was 8.7 months, and OS was 12.3 months. Additionally, the use of chemoimmunotherapy as first-line treatment further improves the survival outcomes of patients with advanced lung cancer.

In this study, the mean age was 69.3 ± 10.7, most of them (79.5%) were male, and older than 70 years (54.5%). These characteristics were consistent with previously published studies [1,13,14]. For example, the patients' characteristics in the Ozturk study were mainly male, with a mean age of 68 years old [14]. Male dominance in this study and Saudi Arabia might be related to increased smoking prevalence or males seeking medical attention more frequently than females, as observed in earlier studies [15,16].

In this study, Adenocarcinoma was commonly observed lung cancer type (79.5%). Our result was similar to a previous study conducted by Ozturk [14]. In this study, the most frequent histologic diagnosis was adenocarcinoma [14]. Additionally, our result was similar to other conducted studies in Saudi Arabia. For example, Albasri evaluated the histopathological analysis of lung cancers and found that adenocarcinoma was the most common pathological type (47.8%), followed by squamous cell carcinoma (25.3%) [15]. The increased prevalence of adenocarcinoma is attributed to improved diagnosis of peripheral pulmonary lesions, revision in WHO cancer classification, and labeling of mucin-producing cells, as well as the impact of atmospheric air pollution, particularly nitrogen oxides which have been linked to an increase in the occurrence of adenocarcinoma lung cancer type [15].

In this study, metastases to the pleura, bones, and mediastinal lymph nodes were the common sites of metastases, which was similar to previous reports from Saudi Arabia [1,6]. One-third of patients, 12 (27.3) in our research had a poor PS at the time of the initial presentation of three or four ECOG classes. This might be attributed to morbidity associated with a more advanced disease state at the time of diagnosis [1].

PD-L1 labeling using classical immunohistochemistry (IHC) is the standard of care (SOC) biomarker for NSCLC patient selection. PD-L1 tumor proportion score (TPS) above 50% could isolate a population of patients in which immunotherapy alone is better than chemotherapy in first-line treatment [12]. In contrast, for other patients, chemo-immunotherapy using platinum-based chemotherapy and anti-PD-1 is the preferred treatment [17]. In recent research in this area, Reck et al. randomized 305 advanced NSCLC patients to pembrolizumab monotherapy versus platinum doublet chemotherapy. Results showed that pembrolizumab improved the main endpoint of progression-free survival (PFS) (median PFS, 10.3 vs. 6 months; hazard ratio (HR): 0.50, 95% confidence interval (CI): 0.37-0.68) and OS compared to chemotherapy (HR: 0.60, 95% CI: 0.41-0.89). The overall risk ratios for pembrolizumab and platinum doublet treatment were 45% and 28%, respectively [18]. In our study, chemotherapy was administered as the first line in 19 cases (43.2) while in 25 (56.8) cases received chemoimmunotherapy. The commonest chemoimmunotherapy regimen used was carboplatin-pemetrexed-pembrolizumab in 16 (36.4%), followed by carboplatin-paclitaxel-pembrolizumab in 9 (20.5%) cases. additionally, our result found that using chemoimmunotherapy as the first-line therapy was associated with numerical survival improvement compared to using chemotherapy alone (HR: 0.75; 95% CI: 0.39-1.46); however, it was not statistically significant (p = 0.397). Similar to our result, Agarwal et al. [19] evaluated 20 patients who received platinum-etoposide and 26 patients who received platinum-etoposide and atezolizumab. Progression-free survival was longer in the chemoimmunotherapy group compared to the chemotherapy group, 4.1 months (95% CI: 3.8-6.9) vs. 3.2 months (95% CI: 0.6-4.8), respectively; p = 0.0491. However, there was no statistically significant difference in the OS between the chemoimmunotherapy and chemotherapy group, 9.3 months (95% CI: 4.9-12.8) vs. 7.6 months (95% CI: 0.6-11.9), respectively; p = 0.21 [19]. In our study, the explanation for nonsignificant survival in chemoimmunotherapy may be attributed to the small sample size. In general, chemoimmunotherapy as a first-line treatment for lung cancer patients with advanced stages prolongs survival and improves the quality of life [19].

In this study, EGFR mutation was positive in two (4.5%) cases and ALK mutation was positive in two (4.5%) cases. The low prevalence of EGFR mutations (2 of our 44) may be attributed to the late introduction of the NGS testing [20,21]. The adenocarcinoma histology of this EGFR-mutated tumor fits well with previous reports preferentially describing mutations in this histologic lung cancer subtype [21,22].

In this study, the median progression-free survival was 8.7 months (IQR: 5.7-11.4), and the median OS was 12.3 months (IQR: 11.1-13.4). The low survival rate in this study was attributed to the advanced age and advanced stage of lung cancer in our patients. Thus, it is difficult to compare OS directly due to the heterogeneous study cohorts, including factors that may affect the prognosis of patients with lung cancer [23]. A previous review study reveals that the overall mortality rate of lung cancer in Saudi patients from 2009 to 2013 was 52.75% (54.82% in SCLC and 50.72% in NSCLC). Tumor stage strongly predicted mortality, with regional extension and distance metastasis increasing mortality by six-fold and five-fold in SCLC, and distance metastasis/systematic disease increasing mortality by three-fold In NSCLC [1]. The lung cancer survival rate shows a difference by histological type, with NSCLC having a better prognosis compared to SCLC. However, the stage of a tumor is the strongest determinant factor of lung cancer survival [1]. Several efforts have tried to develop an accurate prediction model for lung cancer prognosis by adding further factors to tumor staging; although, tumor stage is still the major mortality predictor. Other factors that showed a prognostic effect independent of disease stage include PS, age, and gender [24]. Additionally, several genetic biomarkers associated with lung cancer were found to have a prognostic effect. Other factors like obesity and smoking history were also found to impact survival in lung cancer. Hence, to achieve higher prediction accuracy, a more complex approach that integrates individual, pathological markers, and genetic factors is needed [25]. Our study did not allow for a full investigation of important prognostic factors because of the limited nature of data collected within single-center cancer registries.

Study limitation

The main limitation of the current study was the small number of cases treated in our center and the unavailability of time-to-event data which prevented the use of survival analysis methods and the ascertainment of an accurate estimate of survival rates for each cancer type. For a better characterization of lung cancer mortality, a prediction model that integrates pathological variables, biological markers, and patient physical status is needed. The lack of such factors is assumed to have a residual confounding role in the results. However, we believe that the current results may inspire the adoption of a local initiative for the early detection of lung cancer. Additionally, the monocentric and retrospective design of the study renders it vulnerable to selection and misclassification biases. Furthermore, our result lacks robust statistical analysis and other important factors such as comorbidities, smoking history, and laboratory data which may affect survival outcomes. For that, our result needs to be validated in a large cohort study with strict criteria including multicenter with different levels of facilities.

## Conclusions

In this study, the majority of lung cancer patients were male and over 70 years old. Adenocarcinoma was the most common cancer type. Metastasis to pleura with pleural effusion was the common presentation. The most common treatment used was chemoimmunotherapy with a regimen of carboplatin-pemetrexed-pembrolizumab. Addressing the possible causes of delayed diagnosis of lung cancer is crucial for improved survival outcomes.
